# Dynamics of Gut Microbiota Recovery after Antibiotic Exposure in Young and Old Mice (A Pilot Study)

**DOI:** 10.3390/microorganisms9030647

**Published:** 2021-03-20

**Authors:** Daniel Laubitz, Katri Typpo, Monica Midura-Kiela, Clairessa Brown, Albert Barberán, Fayez K. Ghishan, Pawel R. Kiela

**Affiliations:** 1 Department of Pediatrics, Steele Children’s Research Center, University of Arizona, 1501 N. Campbell Ave, Tucson, AZ 85724, USA; laubitz@email.arizona.edu (D.L.); ktyppo@arizona.edu (K.T.); mkiela@peds.arizona.edu (M.M.-K.); 2 Department of Environmental Science, University of Arizona, 1657 E. Helen St., Tucson, AZ 85721, USA; clairessabrown@gmail.com (C.B.); barberan@arizona.edu (A.B.); 3 Department of Immunobiology, University of Arizona, 1656 E. Mabel St., Tucson, AZ 85724, USA

**Keywords:** aging, bacteria, 16S, antibiotics, metronidazole, ciprofloxacin

## Abstract

Antibiotics have improved survival from previously deadly infectious diseases. Antibiotics alter the microbial composition of the gut microbiota, and these changes are associated with diminished innate immunity and decline in cognitive function in older adults. The composition of the human microbiota changes with age over the human lifespan. In this pilot study, we sought to identify if age is associated with differential recovery of the microbiota after antibiotic exposure. Using 16S rRNA gene sequencing, we compared recovery of the gut microbiota after the 10-day broad-spectrum antibiotic treatment in wild-type C57BL/six young and older mice. Immediately after antibiotic cessation, as expected, the number of ASVs, representing taxonomic richness, in both young and older mice significantly declined from the baseline. Mice were followed up to 6 months after cessation of the single 10-day antibiotic regimen. The Bray-Curtis index recovered within 20 days after antibiotic cessation in young mice, whereas in older mice the microbiota did not fully recover during the 6-months of follow-up. *Bifidobacterium*, *Dubosiella*, *Lachnospiraceae*_NK4A136_group became dominant in older mice, whereas in young mice, the bacteria were more evenly distributed, with only one dominant genus of *Anaeroplasma.* From 45 genera that became extinct after antibiotic treatment in young mice, 31 (68.9%) did not recover by the end of the study. In older mice, from 36 extinct genera, 27 (75%) did not recover. The majority of the genera that became extinct and never recovered belonged to *Firmicutes* phylum and *Clostridiales* family. In our study, age was a factor associated with the long-term recovery of the gut microbiota after the 10-day antibiotic treatment.

## 1. Introduction

The microbial composition of a mature human gut was thought to develop in the first few years of life and remain relatively stable throughout adulthood [1]. However, recent studies have shown that as humans age, distinct gut microbiota profiles emerge that differentiate infants from older children and younger adults, and middle-aged adults from elderly adults [2,3,4]. Shifts in microbial community structure evident in elderly adults may have both acute and long-term functional consequences as these shifts are associated with diminished innate immunity, overall frailty, and diminished cognitive function [5,6,7,8]. In turn, shifts in microbiota community structure during infancy are associated with the development of several chronic adult illnesses, including diabetes, asthma, and metabolic syndrome [9,10,11,12,13].

Many sociological and medical factors coincide with aging, including changes in diet, living and socializing arrangements, the presence of chronic illnesses, and the use of medications that may trigger or promote these age-associated microbial community changes [5,6]. This makes evaluating host-related factors responsible for shaping the gut microbiota during human aging challenging. Differences in the microbial community structure reported in elderly individuals have been primarily attributed to dietary changes, which include a less varied diet consisting of low fiber-containing foods [5]. In the elderly, the antibiotic (Abx) treatment is also associated with a rapid loss of microbiota diversity.

This decrease in microbiota diversity in combination with dietary changes and Abx treatment in institutionalized elderly individuals is associated with negative effects to the patient such as elevated systemic inflammation indices, depression, and general frailty [5]. In infants, the Abx treatment also results in a rapid decrease in microbiota diversity with subsequent recovery of the microbiota. However, these transient shifts may have permanent consequences to immune and metabolic programming in infants, as the interaction of the microbiota with the host affects the developing immune system and metabolism and may be implicated in the development of life-long chronic illnesses [11,14,15,16,17,18].

While external environmental factors are critical to the composition of gut microbiota, molecular signals within the host gut epithelium associated with aging are also important [19]. During aging, microbes may alter host inflammatory and immune functions through gut-microbial crosstalk, which in turn could feed-back to changes to microbial species composition and function [20,21]. As humans age, the gut microbiota shifts from bacterial populations with immune-modulating functions to one with overrepresentation of pathobionts [5,22,23]. Normal age-associated decline in immune surveillance functions might promote the pathobiont niche, especially after treatment with Abx. Our objective was to conduct a pilot experiment to assess the dynamics of microbiota recovery after broad spectrum Abx treatment in young and middle-aged mice. This study sought to understand the structural and functional consequences of broad-spectrum Abx utilization on the recovery of the gut microbiota in two age epochs.

## 2. Materials and Methods

### 2.1. Mice and Antibiotic Treatment

Our objective was to study the short-term and long-term effect of the 10-day broad spectrum antibiotic treatment on the microbiota population in the mouse gut in both young and older mice. All of the mice used in this work were kept in the University of Arizona Animal Care, Specific Pathogen Free facility, and handled in accordance with the university’s guidelines and with an approved IACUC protocol [Kiela, 07-126]. All of the mice had ad libitum access to food and 12/12 h day/night cycle.

**Figure 1:**
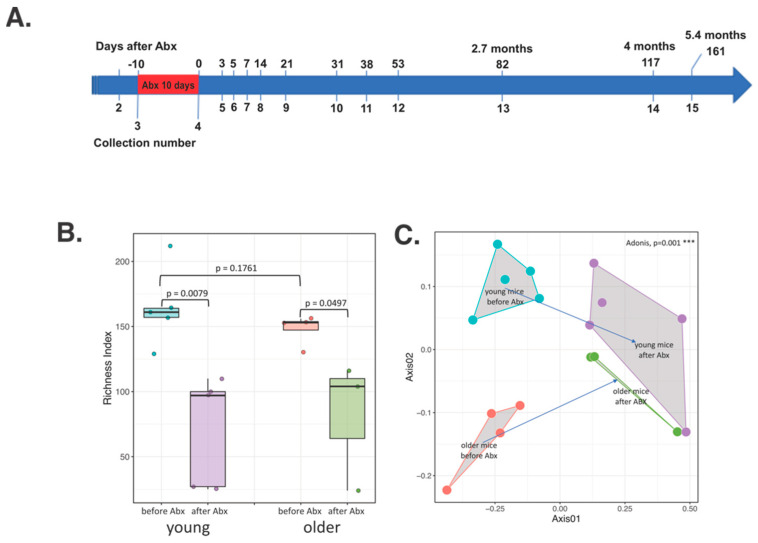
(**A**) Sampling and antibiotic treatment schematic for 6 months. All of the mice were treated with the antibiotic cocktail for 10 days (red) and stool samples were collected as shown by the collection numbers. The numbers above the time scale depict the day since the antibiotic treatment, whereas the corresponding collection number below depict when the samples were collected. (**B**) Box plot of amplicon sequence variant (ASV) richness in young and older mice before and after 10 days of antibiotic treatment. Points represent the ASV richness of each mouse gut microbiota sample. *P*-values were calculated with the Mann-Whitney test. (**C**) Bray-Curtis based non-metric multidimensional scaling (NMDS) plot of distances between young and older mice before and after antibiotic treatment.

Five 7-week old and five 40-week old SPF C57BL/six female mice originating from the same colony were treated with broad-spectrum antibiotics metronidazole (500 mg/L) and ciprofloxacin (200 mg/L) (Abx, both from Alfa Aesa, Haverhill, MA, USA) for 10 days in drinking water to simulate broad spectrum antibiotic utilization in humans. Supplemented water was provided *ad libitum* and checked daily. Previously, we have described a sexual dimorphism in response to this antibiotic treatment [24]. Therefore, for this study, we chose only female mice to avoid confounding by sex. Seven week and 40-week mice are respectively equivalent to 5-year-old and middle-aged human adults and were therefore representative of our age groups of interest. Fecal pellets were collected from all of the mice for 7-days prior to the treatment with antibiotics, as well as on the day of antibiotic initiation. After 10 days of antibiotic supplementation into drinking water, the mice were switched back to antibiotic-free, autoclaved drinking water and fecal pellets were collected over 6 months, as shown in Figure 1A. All of the fecal samples were kept at –80 °C. Microbial genomic DNA from all of the samples was purified with PowerFecal Pro DNA kit (Qiagen, Germantown, MD, USA; Cat no. 51804) according to the manual provided by the manufacturer. The samples were homogenized using the provided lysis buffer and the tubes were pre-filled with the 96-well plate shaker (Mo-Bio, now Qiagen, Germantown, MD, USA; Cat no. 11996) with 2 mL adapters (Mo-Bio, Cat no. 11990), two times for 10 min at a speed of 30 Hz each at 4 °C.

### 2.2. Gut Microbiota Analysis

The hypervariable V4 region of the 16S rRNA gene was amplified from each sample using unique barcoded reverse primers (806R), the same for each sample forward primer (515F), and MyFi^TM^ Mix (Bioline Meridian, Memphis, TN, USA; Cat no. BIO-25050). Both reverse and forward primers are extended with the sequencing primer pads, linkers, and Illumina adapters [25]. The PCR was performed on LightCycler 96 (Roche) in the final volume 40 μL. The PCR conditions were as follows: Initial denaturation at 95 °C for 120 s followed by 35 cycles of 95 °C for 30 s, 50 °C for 30 s, and 72 °C for 30 s, with the final elongation at 72 °C for 300 s. Amplicons were quantified using the Quant-It PicoGreen dsDNA Assay kit (Thermo Fisher Scientific, Waltham, MA, USA; Cat no. P7589), according to the manufacturer’s protocol. Equal amounts of amplified DNA (240 ng) from each sample were pooled and cleaned using the UltraClean PCR Clean-Up kit (MoBio, now Qiagen, Germantown, MD, USA; Cat no. 12500). Pooled amplicons were diluted, denatured with NaOH at a final concentration of 0.1 N, and 6.75 pmols of the pooled library were sequenced at our laboratory on the MiSeq platform (Illumina) using custom primers [25]. Due to the limited sequence diversity among 16S rRNA amplicons, 5% of the PhiX Sequencing Control V3 (Illumina, San Diego, CA, USA; Cat no. FC-110-3001) made from phiX174, was added to the run. The pooled 16S rRNA library was subjected to the paired-end sequencing using 2 × 150 bp MiSeq Reagent kit v2 (Illumina, San Diego, CA, USA; Cat no. MS-102-2002). The length of the sequences after merging was 232–233 bp with a median overlapping fragment of 27 bp (min 26 bp, max 29 bp). De-multiplexing was done using the *idemp* script (https://github.com/yhwu/idemp; accessed on 20 November 2020). Filtering, dereplication, sample inference, chimera identification, and merging of paired-end reads was done with a reference-free Divisive Amplicon Denoising Algorithm 2 (*Dada2,* version 1.16.0) in RStudio (version 1.3.959 with R version 4.0.2) package [26]. The ASVs taxonomy was assigned using the RDP classifier against SILVA database release 132 [27] (https://www.arb-silva.de/documentation/release-132/; accessed on 20 November 2020). The *vegan* package (version 2.5.6) [28] was used as a tool for diversity analysis, ordination methods, for the analysis of dissimilarities, and statistical analysis. The obtained results were visualized with *ggplot2* package (version 3.3.2) [29]. The linear discriminant analysis effect size (LEfSe, Galaxy platform) was used to determine the different ASVs between young and older mice before and after the antibiotic treatment by coupling standard tests for statistical significance with additional tests encoding biological consistency and effect relevance [30]. Differential abundance of taxa between groups were calculated using the unrarefied count table with DESeq2 (R package version 1.28.1) [31], and for statistical analysis the Wald test was used and *p*-values were corrected with the Benjamini-Hochberg method.

The total number of sequenced samples was 378, however, only 177 samples belong to this study. After filtering out all of the unwanted samples, 177 samples were left. After QC filtering and removing chimeras, the average number of reads per sample was 36,419 (min = 6489, max = 98,258). To even the sampling depth, all of the samples were rarified at the 6489 reads. Sequences for all of the samples were submitted to and deposited in the NCBI sequence read archive (SRA) under accession reference PRJNA667480.

## 3. Results

### 3.1. Short-Term Gut Microbiota Alterations after Broad-Spectrum Abx Treatment

Short-term changes in the bacterial community structure as a response to the Abx treatment have been previously described. [32,33,34,35,36] Here, we compare the short- and long-term differences in the gut microbiotas of young and older mice after the broad spectrum Abx treatment. Young (4 week-old) and older (9 month-old; *n* = 5 each) mice obtained from the same colony of wild-type C57BL/six mice kept in separate cages were treated with metronidazole and ciprofloxacin for 10 days and the fecal pellets were collected prior to and on the final day of Abx administration and for up to 6 months of follow-up (Figure 1A).

At the onset of the study (before the Abx treatment), older mice tended to have a lower amplicon sequence variant (ASV) richness, with significantly decreased Simpson index (lower evenness), and decreased Shannon index, although the latter did not reach statistical significance (Figure S1A). Bray-Curtis based non-metric multidimensional scaling (NMDS) revealed significantly different (ADONIS test, *p* = 0.006) microbial communities between young and older mice prior to the Abx treatment (Figure S1B). DESeq2 analysis revealed significant changes (adjusted *p* < 0.05) in the taxonomic composition in young and older mice, which are depicted in Figure S2 and Table S1. Among the genera with age-related increase in relative abundance were Ruminoclostridium_6, Lachnospiraceae_NK4A136_group, Erysipelatoclostridium, Faecalibaculum, and Anaeroplasma, while a significant age-related decrease was documented for Candidatus_Stoquefichus, Candidatus_Arthromitus (segmented filamentous bacteria, SFB), Butyricicoccus, Ruminoclostridium_6, Lachnoclostridium, Oscillibacter, Roseburia, Lactobacillus, Ruminococcaceae_UCG-014, and Parabacteroides (Figure S2B,C, Supplementary Data File S1).

**Figure 2:**
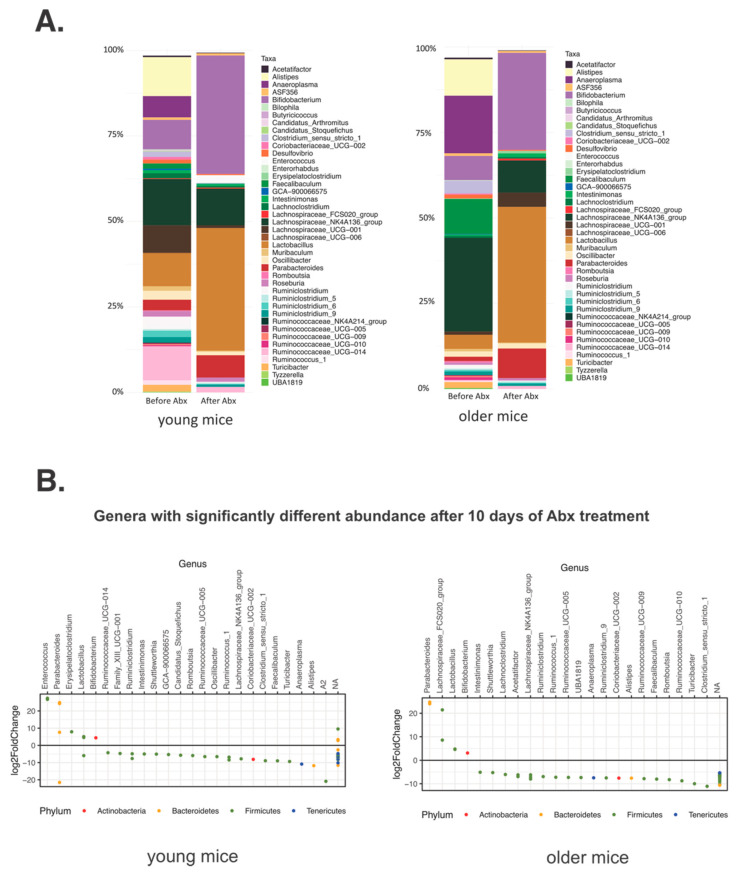
Changes in taxonomical composition after the 10-day treatment with Abx cocktail at genus level in (**A**) young and older mice. Genera with relative abundance lower than 0.5% were removed from the graphs for clarity. (**B**) The genus level differential abundance analysis with DESeq2 showing changes in taxa abundance in young (left panel) and older (right panel) mice after the Abx treatment. Each dot represents the log2 fold change between “before” and “after” the antibiotic treatment.

Immediately after Abx cessation, as expected, the number of ASVs, representing taxonomic richness, in both young and older mice significantly declined from the baseline (Figure 1B). Additional measures of alpha diversity as well as the Shannon and Simpson Indices, were also decreased, albeit more significantly in younger mice (Figure S3). These latter two indices representing richness/evenness and richness/relative abundance (dominance) respectively, had lower values in older mice at the baseline, which may have impacted the magnitude of change. Non-metric multidimensional scaling (NMDS) on Bray-Curtis-calculated distances showed that microbial community changes in both groups had the same direction and that the remaining bacterial communities after the Abx treatment were more similar between the two age groups than prior to the Abx treatment (Adonis, R = 0.58775, *p* = 0.001) (Figure 1C). The Abx treatment resulted in the declining relative abundance of majority of the microbial taxa in the gut, and very similar results of the taxonomic composition between age groups (Figure 2A,B). When the taxonomic composition before and after the Abx treatment was compared in older and young mice, we identified different genera significantly declining in older and young mice at the conclusion of Abx treatment, as shown by the DESeq2 analysis (Figure 2B, Supplementary Data File S2). This suggested that the Abx treatment altered the microbiota in both age groups in disparate ways, but resulted in a similar, new baseline (Figure 2B). During the treatment with broad-spectrum Abx, the relative abundance of nearly all bacterial taxa declined. The lists of decreased genera in both younger and older mice were very similar, with many taxa no longer detected (Figure 2, Tables S2 and S3). The relative abundance of *Lactobacillus*, *Bifidobacterium*, *Parabacteroides*, *Ruminococcaceae*_UCG-014 were increased after the Abx treatment, likely as a reflection of their relative resistance to the used antibiotics (Figure 2B, Tables S2 and S3).

### 3.2. Differential Long-Term Recovery from a Single Abx Treatment of Young and Older Mice

**Figure 3:**
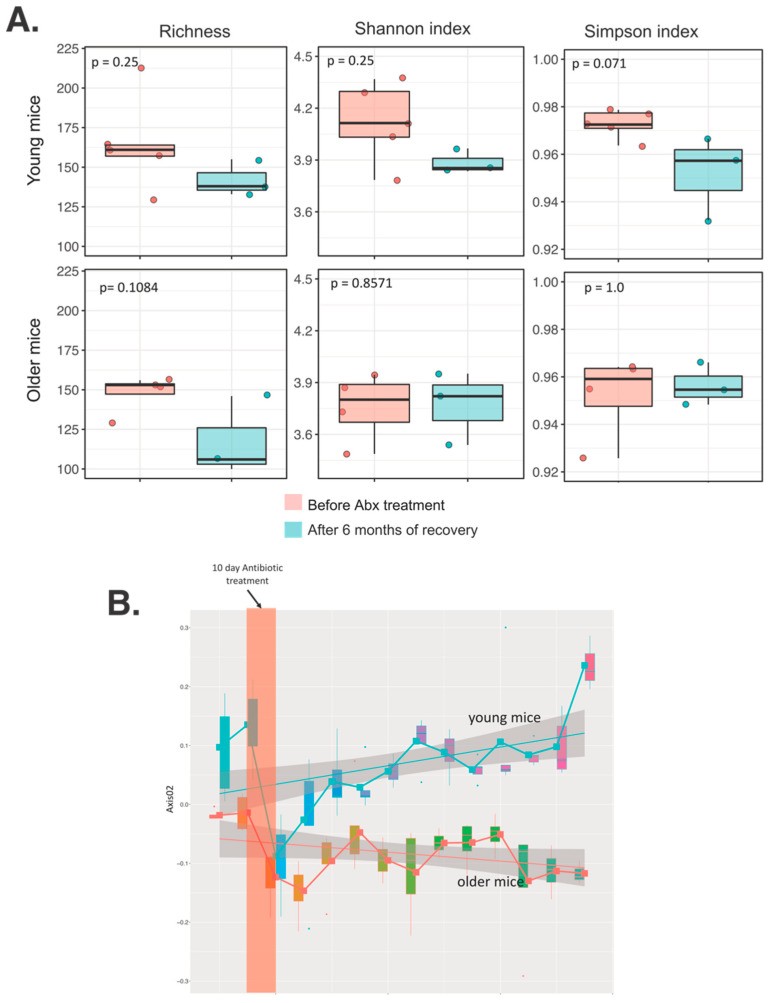
Long-term effect of a single broad-spectrum Abx cocktail treatment on (**A**) alpha diversity indices and (**B**) Bray-Curtis dissimilarity in gut microbiota of young and older mice.

Mice were followed up to 6 months after cessation of the single 10-day Abx regimen to determine the dynamics of the gut microbiota restoration. In both young and older mice, the Richness index tended to be lower at the end of the experiment compared to the values before the treatment (Figure 3A). Surprisingly, Shannon and Simpson indices recovered in older mice to the base level, however, in younger mice these two indices remained lower, albeit without reaching a statistical difference. Since both Simpson and Shannon indices incorporate richness and the number of taxa (dominance) or evenness, respectively, the results indicate that older mice, although having an overall fewer number of taxa, had a few more highly abundant taxa remaining. Bray-Curtis, a non-phylogenetic dissimilarity metric, showed a divergence between young and older mice over time during the recovery period (Figure 3B). The Bray-Curtis index recovered within 20 days after Abx cessation in young mice, whereas in older mice microbiota did not fully recover during the 6 months of follow-up.

**Figure 4:**
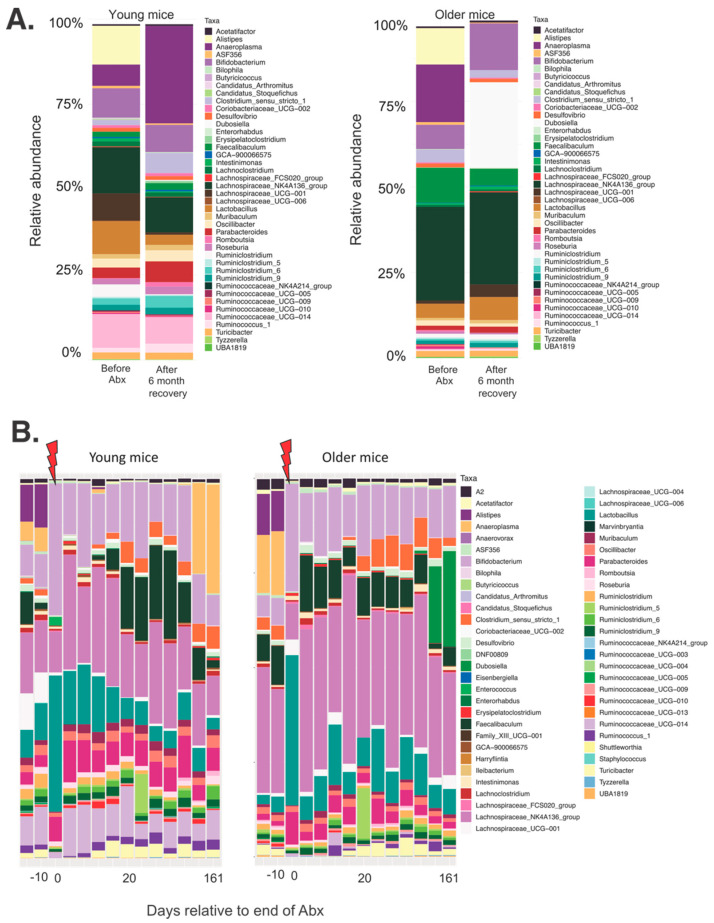
Changes in the distribution of abundant genera in fecal samples from young and older mice (**A**) before Abx and 6 months after the Abx treatment, and (**B**) all of the collections timepoints. Genera with relative abundance lower than 0.5% were removed from the graphs for clarity.

*Bifidobacterium*, *Dubosiella*, *Lachnospiraceae*_NK4A136_group became dominant in older mice, whereas in young mice, the bacteria were more evenly distributed, with only one dominant genus of *Anaeroplasma* (Figure 4).

**Figure 5:**
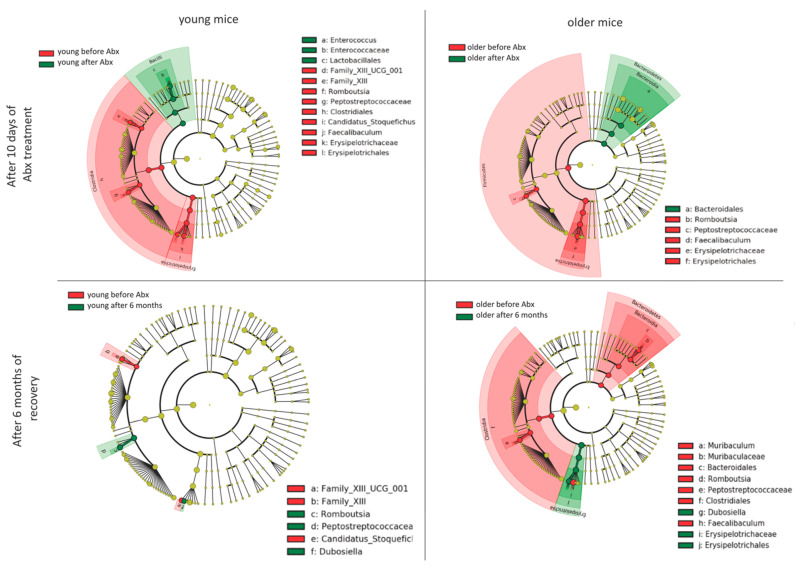
Linear discriminant analysis (LDA) effect size (LEfSe) of differentially abundant bacterial taxa between young (left panels) and older mice (right panels) after 10 days of Abx treatment (upper panels) or after 6 months of recovery (lower panels). Cladograms represent phylogenetic branches of taxa significantly more abundant in the two analyzed groups.

To determine taxa (ASVs) explaining both short- and long-term differences between young and older mice recovering from the Abx treatment, we applied the linear discriminant analysis (LDA) effect size (LEfSe) method (Figure 5). In young mice, the short-term (10 days of Abx) differences were mostly driven by the lower abundance of genera *Feacalibaculum* and *Candidatus_Stoquefichus* both from family *Erysipelotrichaceae*, as well as genus *Romboutsia* from family *Peptostreptococcaceae*, and genus family_XIII_UCG_001, a member of the *Family_XIII*. Both *Peptostreptococcaceae* and *Family_XIII* families are members of the class *Clostridia*, phylum Firmicutes. We also observed an increased relative abundance of *Enterococcus* genus. In older mice, the LEfSe analysis showed similar changes, however, the increased relative abundance of *Bacteroidales* was observed 10 days after Abx cessation. When the LEfSe algorithm was applied to compare taxa in young mice before the Abx treatment and after 6 months of recovery, genera *Rombustia* and *Dubosiella* were enriched and Famliy_XIII_UCG_001 and *Candidatus_Stoquefichus* decreased. However, those changes were limited mostly to genus and family levels with no detectable changes in higher taxonomic hierarchy. In older mice, long-term recovery was associated with a reduced relative abundance of a class of Clostridia (phylum Firmicutes), driven primarily by the genus *Romboutsia,* and the expansion of the class *Bacteroidetes*, driven primarily by the genus *Muribaculum.* In contrast, *Dubosiella*, a member of *Erisipelotrichaceae* family was enriched in older mice after long-term recovery.

We also analyzed the long-term recovery of bacterial genera most severely affected by the Abx treatment, i.e., brought to below the detection limit, or “extinct” immediately after the Abx cessation (the complete results of DESeq2 analysis are provided in Supplementary Data File S3). From 45 genera that became extinct in Abx-treated young mice, 31 (68.9%) did not recover by the end of the study. In older mice, from 36 extinct genera, 27 (75%) did not recover. The Venn diagram analysis showed 21 genera that faced long-term extinction in both age groups, nine genera that remained extinct only in younger mice and three genera that remained extinct in older mice (Figure S4). The majority of the genera that became extinct and never recovered belonged to *Firmicutes* phylum and *Clostridiales* family.

## 4. Discussion

Antibiotics are commonly used as a life-saving treatment for previously fatal infectious diseases. However, due to their availability, patient demand, and relatively low cost, antibiotics are often inappropriately prescribed, which has had a dramatic negative impact on the gut microbiota and emergence of antibiotic-resistant bacterial species [37,38,39,40,41]. Different antibiotics have varied mechanisms of action and therefore, have a differential impact on resident gut microbial populations [37]. In our study, we found that age was also a factor associated with a differential recovery of the intestinal microbiota after a 10-day exposure to the broad-spectrum Abx treatment. In the short-term, Abx resulted in a decrease in the overall number of taxa, and in particular, a decrease in the relative abundance of Firmicutes in both age groups. The age-related differences in microbial composition apparent at the baseline were no longer apparent and the overall community composition was similar immediately after the Abx treatment, thus allowing us to infer the role of the host’s age on the qualitative community recovery. The long-term impact of Abx treatment differed between the age groups. Mice that were older at the start of our experiment had only partial recovery of their pre-Abx baseline by 6 months. Mice that were younger at the start of our experiment showed a more complete recovery of their baseline microbiota within 20 days after the Abx treatment.

The antibiotic treatment typically reduces the overall bacterial load in the gut, but also results in shifts in the relative abundance of the remaining taxa, as seen in our study. Microbial population analysis based on 16S rRNA amplicon profiling allows identification and analysis of the relative abundance of individual taxa. This caveat can lead to misinterpretation of results when the relative abundance of one or more taxa declines while the remaining taxa increase. This “virtual” increase is a result of the dramatically decreased abundance of other groups, therefore the proportion of those which can respond slower to this particular antibiotic mixture are increased. This phenomenon can lead to false conclusions, that a taxon is resistant to antibiotics or they overgrow in the presence of antibiotics. Rather, these taxa became “dominant” within the depleted gut bacterial community. We observed this phenomenon in our data, represented by changes in the relative abundance of microbial populations after the antibiotic treatment. However, the 16S rRNA amplicon profiling approach remains valuable in assessing the recovery of the complex microbial community from an Abx insult over time.

Aging is associated with a shift in the microbiota from a population with immune modulatory functions to an increase in abundance of pathobionts [5,22,23]. The antibiotic treatment variably decreases microbial populations, and in the setting of an aging host, it may result in an altered microbiota in the long-term [22]. Our study is consistent with prior reports of youth-associated and aging-associated microbial populations. Youth associated microbiota have an increased relative abundance of bacteria with immune-modulating functions, while aging-associated microbiota have an increase in the relative abundance of pathobionts and a decrease in bacterial populations with immune-modulating properties [5,22,23]. In our study, this trend was seen when we compared younger and older age groups, representing school-aged children and middle-aged adults. For example, younger mice, as compared to older mice had an increased relative abundance of *Roseburia*, a gut symbiont which promotes and regulates immunity [42]. It is notable, that the younger age group mice in our study reach the age of our older mice at the end of our long-term antibiotic observation period. However, the microbiota composition of our young mice post-Abx treatment did not mirror the baseline microbiota of the older mice observed at the start of our experiment. This suggests that while the young mice recovered to their prior baseline microbiota after 6 months, that antibiotics disrupted the normal evolution of the microbial community that occurs with aging. The interpretation of this pilot data needs obvious scrutiny. While the longitudinal design of the study allows us to attribute the observed differences to age, a more extensive study is certainly justified. In such an experiment, aging mice originating from the same cohort at birth, with sex and cage as confounding variables, as well as a parallel negative control group without antibiotics would be needed to fully address the posed questions.

Aging is also associated with a decline in immune surveillance functions, and it is possible that the host-microbial interface in the setting of less effective host immune surveillance, results in incomplete recovery of the host microbiota [19,43]. The symbiotic relationship between the host physiology and microbes appears disturbed by the antibiotic use and the aging-associated changes to the immune function, surveillance, and the intestinal epithelium further resulting in a microbiota that is unable to fully recover [44], as observed in our study. The decline in immune surveillance that normally occurs with age, may allow these pathobiont niches to persist after the antibiotic treatment. The persistence of pathobionts and loss of commensal organisms may increase the risk for older individuals for acute bacterial or fungal infections, with significant impact on longevity and/or quality of life. A currently ongoing clinical trial (ClinicalTrials.gov Identifier: NCT04171466) plans to investigate the effects of the same set of antibiotics (Ciprofloxacin + Metronidazole) and the recovery of the gut microbial communities with and without a multi-strain symbiotic SH-DS01 in patients 18–55 years old. Our data suggest that this and other similar studies should strongly consider the patient’s age in data analysis and the assessment of treatment efficacy.

## Data Availability

Sequences for all of the samples were submitted to and deposited in the NCBI sequence read archive (SRA) under accession reference PRJNA667480.

## Supplementary Materials

The following are available online at https://www.mdpi.com/2076-2607/9/3/647/s1. Figure S1. Box plots of Richness, Shannon and Simpson indices. Figure S2. (A) Relative abundance of genera with relative abundance >0.5% in young and older mice at the baseline. (B) Bar graph of differentially abundant genera in older mice compare to the relative abundance in young mice (fold change). C) Differential abundance analysis (DESeq2) of genera in older and young mice. The dots represent statistically significant (Benjamini-Hochberg adjusted *p* < 0.05) the Log2 Fold Change of genera in older mice in comparison to young mice (bold line). Figure S3. Box plots of Shannon and Simpson indices in young and older mice before and after 10 days of Abx treatment. Figure S4. Venn diagram and the corresponding table depicting the number and classification of the genera that remained extinct at the time of and 6 months after the cessation of Abx treatment in young and/or older mice. Table S1. List of the most abundant genera in fecal samples of young and older mice. Table S2. List of the most abundant genera in fecal samples of young mice before and after 10-day broad-spectrum antibiotic treatment. Table S3. List of the most abundant genera in fecal samples of older mice before and after 10-day broad-spectrum antibiotic treatment. Supplementary data file S1: older vs young mice before Abx. Supplementary data file S2: older mice 10days after Abx and young mice 10days after Abx. Supplementary data file S3: older mice recovery 6mo and young mice recover 6 mo.
