# Supplementary material for: Dynamics of Gut Microbiota Recovery after Antibiotic Exposure in Young and Old Mice (A Pilot Study)
Source: Microorganisms. 2021 Mar 20;9(3):647. doi: 10.3390/microorganisms9030647 (PMC8003781; doi:10.3390/microorganisms9030647)
Supplement: Supplementary file 1 [file Figure S2.pdf › Figure S2.pdf]

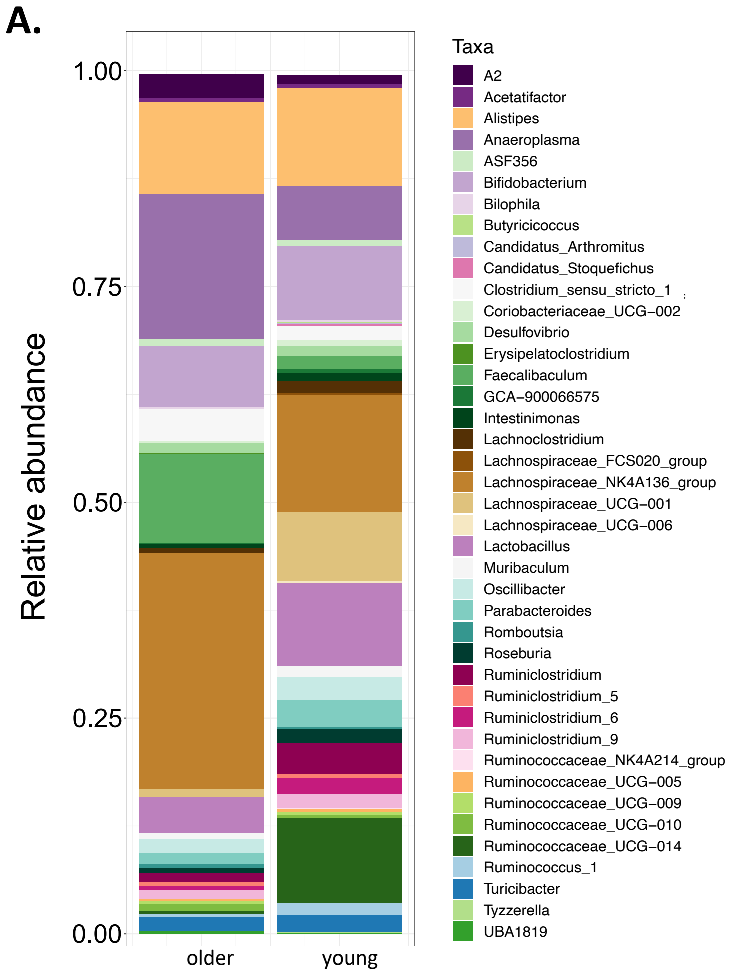

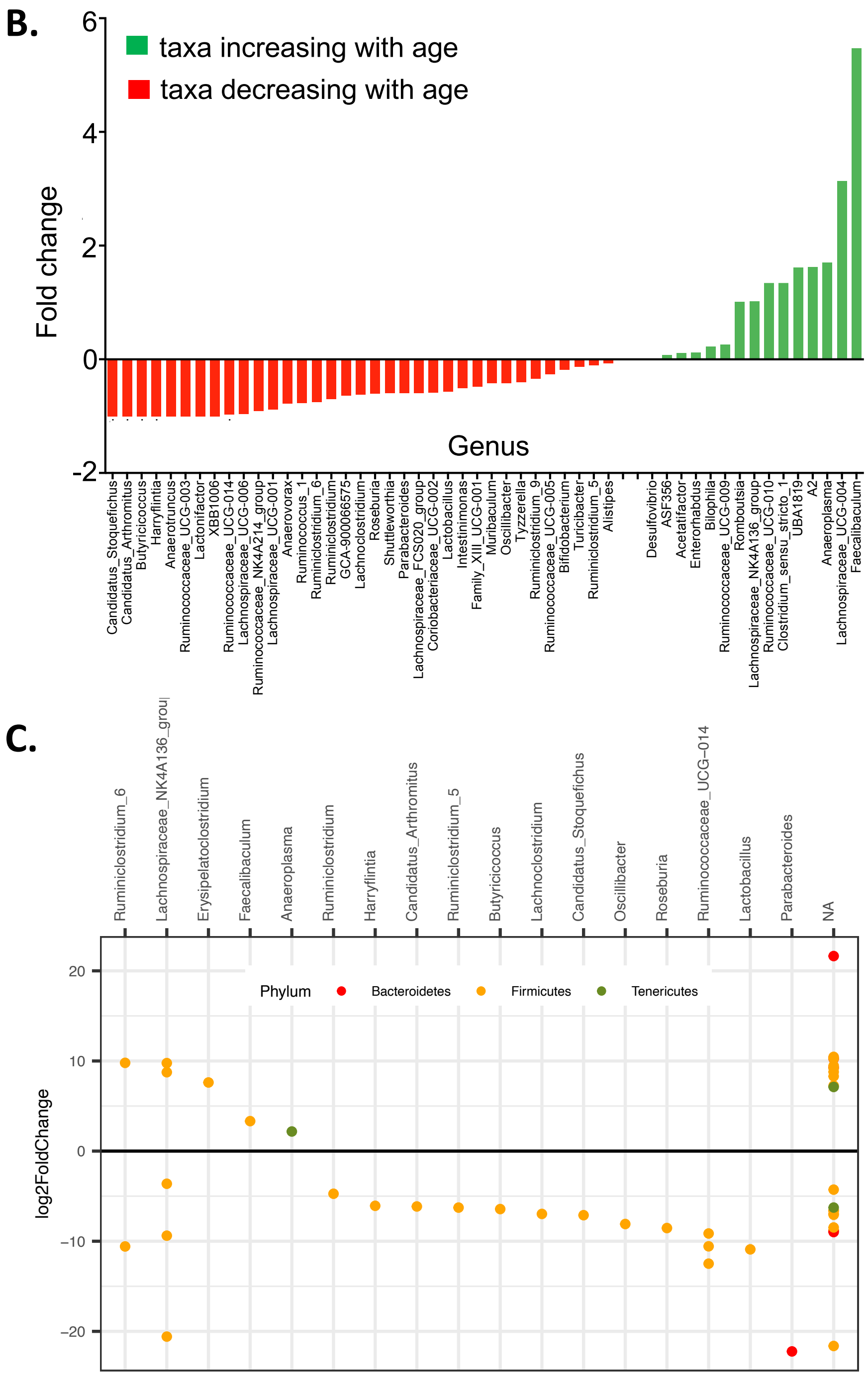

Figure S2. (**A**) Relative abundance of genera with relative abundance >0.5% in young and older mice at the baseline. (**B**) Bar graph of differentially abundant genera in older mice compare to the relative abundance in young mice (fold change). C) Differential abundance analysis (DESeq2) of genera in older and young mice. The dots represent statistically significant (Benjamini-Hochberg adjusted $p < 0.05$) the Log2 Fold Change of genera in older mice in comparison to young mice (bold line).
