# Supplementary material for: Dynamics of Gut Microbiota Recovery after Antibiotic Exposure in Young and Old Mice (A Pilot Study)
Source: Microorganisms. 2021 Mar 20;9(3):647. doi: 10.3390/microorganisms9030647 (PMC8003781; doi:10.3390/microorganisms9030647)
Supplement: Supplementary file 1 [file microorganisms-09-00647-s001.zip › Figure S1.pdf]

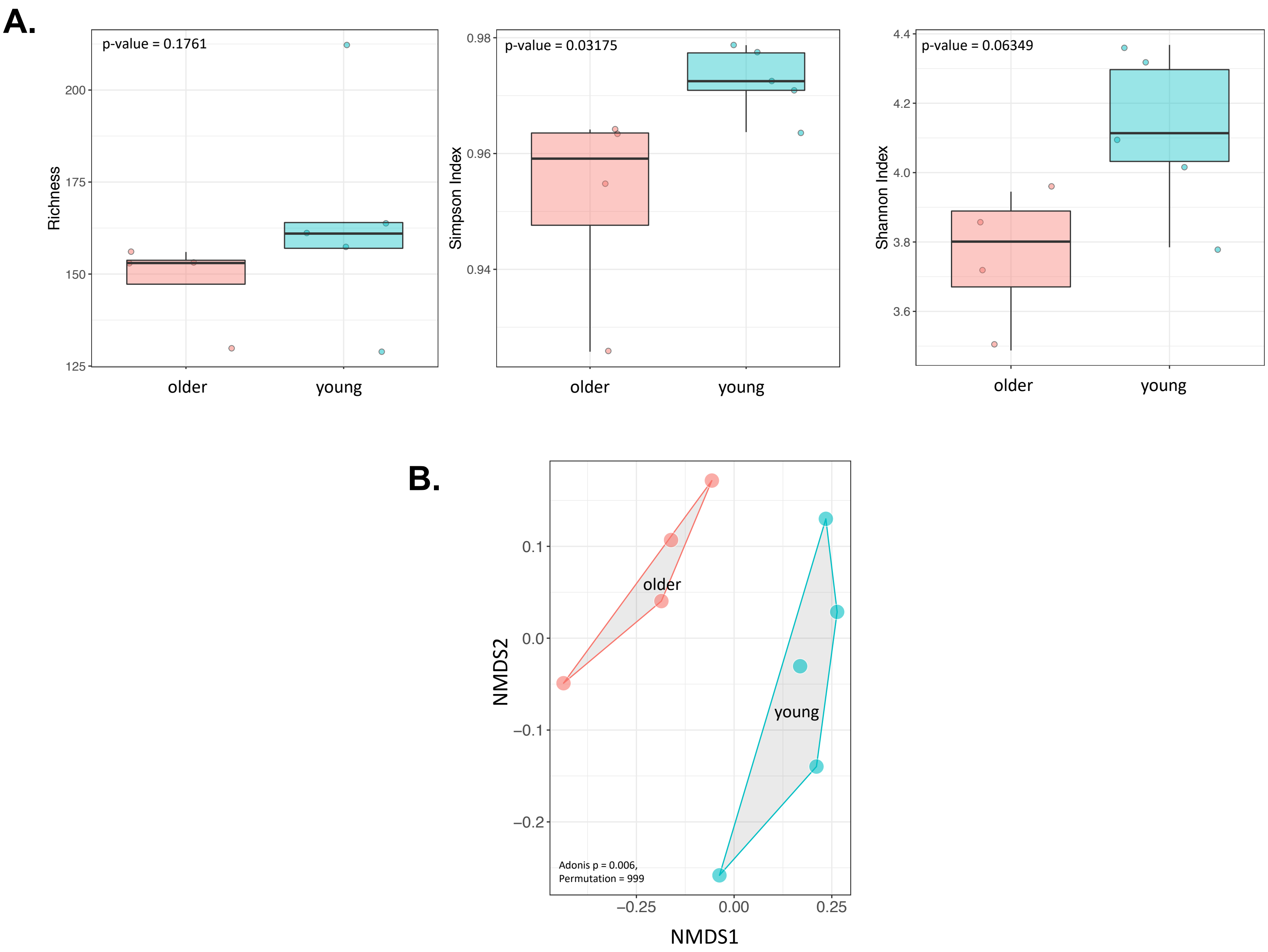

Figure S1. Box plots of Richness, Shannon and Simpson indices (**A**) and Bray-Curtis dissimilarity based NMDS (**B**) comparison between fecal microbiome in young and older mice at baseline. Each dot represents an individual mouse. The lower and upper hinges represent the first and third quartiles respectively. The whiskers indicate min-max values. The bolded midline represents the median. Alpha diversity indices for young and older mice were compared with Mann-Whitney test.
