# Supplementary material for: Dynamics of Gut Microbiota Recovery after Antibiotic Exposure in Young and Old Mice (A Pilot Study)
Source: Microorganisms. 2021 Mar 20;9(3):647. doi: 10.3390/microorganisms9030647 (PMC8003781; doi:10.3390/microorganisms9030647)
Supplement: Supplementary file 1 [file microorganisms-09-00647-s001.zip › Figure S3.pdf]

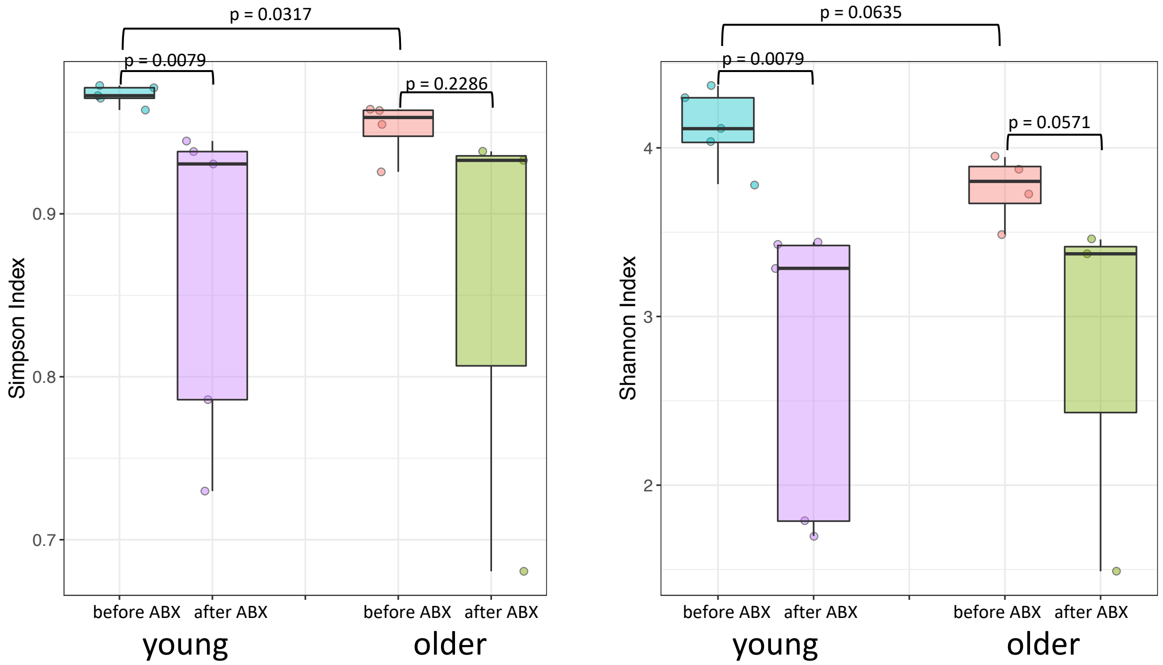

Figure S3. Box plots of Shannon and Simpson indices in young and older mice before and after 10 days of Abx treatment. Each dot represents an individual mouse. The lower and upper hinges represent the first and third quartiles respectively. The whiskers indicate min-max values. The middle line represents the median. P values calculated with Mann-Whitney test.
