# Supplementary material for: Dynamics of Gut Microbiota Recovery after Antibiotic Exposure in Young and Old Mice (A Pilot Study)
Source: Microorganisms. 2021 Mar 20;9(3):647. doi: 10.3390/microorganisms9030647 (PMC8003781; doi:10.3390/microorganisms9030647)
Supplement: Supplementary file 1 [file microorganisms-09-00647-s001.zip › Figure S4.pdf]

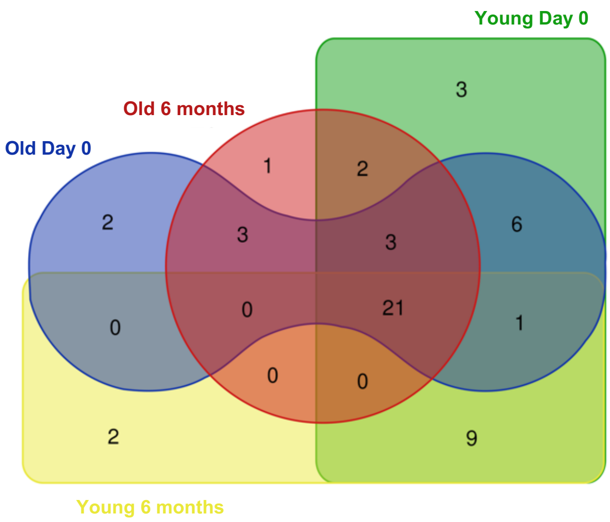

Description	Total	Classification
long-term extinction in both age groups	21	*Firmicutes_Clostridia_Clostridiales_Lachnospiraceae_Lachnospiraceae_NK4A136_group*<br>*Firmicutes_Clostridia_Clostridiales_Lachnospiraceae_NA*<br>*Firmicutes_Clostridia_Clostridiales_Ruminococcaceae_Oscillibacter*<br>*Firmicutes_Clostridia_Clostridiales_Ruminococcaceae_Ruminococcaceae_UCG-014*<br>*Firmicutes_Clostridia_Clostridiales_Family_XIII_NA*<br>*Firmicutes_Clostridia_Clostridiales_NA_NA*<br>*Firmicutes_Clostridia_Clostridiales_Ruminococcaceae_Ruminiclostridium_5*<br>*Firmicutes_Clostridia_Clostridiales_Peptococcaceae_NA*<br>*Firmicutes_Clostridia_Clostridiales_Family_XIII_Anaerovorax*<br>*Firmicutes_Clostridia_Clostridiales_Lachnospiraceae_GCA-900066575*<br>*Firmicutes_Clostridia_Clostridiales_Family_XIII_Family_XIII_UCG-001*<br>*Actinobacteria_Coriobacteriia_Coriobacteriales_Eggerthellaceae_Enterorhabdus*<br>*Firmicutes_Clostridia_Clostridiales_Ruminococcaceae_Ruminiclostridium*<br>*Tenericutes_Mollicutes_Mollicutes_RF39_NA_NA*<br>*Bacteroidetes_Bacteroidia_Bacteroidales_Muribaculaceae_NA*<br>*Firmicutes_Clostridia_Clostridiales_Lachnospiraceae_Lachnospiraceae_UCG-006*<br>*Firmicutes_Clostridia_Clostridiales_Clostridiales_vadinBB60_group_NA*<br>*Firmicutes_Clostridia_Clostridiales_Lachnospiraceae_Lachnoclostridium*<br>*Firmicutes_Clostridia_Clostridiales_Ruminococcaceae_NA*<br>*Firmicutes_Clostridia_Clostridiales_Lachnospiraceae_Acetatifactor*<br>*Firmicutes_Clostridia_Clostridiales_Ruminococcaceae_Intestinimonas*
long-term extinction in older mice ONLY	3	*Firmicutes_Clostridia_Clostridiales_Ruminococcaceae_Ruminococcaceae_UCG-010*<br>*Firmicutes_Clostridia_Clostridiales_Eubacteriaceae_Anaerofustis*<br>*Actinobacteria_Coriobacteriia_Coriobacteriales_Eggerthellaceae_DNF00809*
long-term extinction in young mice ONLY	9	*Firmicutes_Clostridia_Clostridiales_Lachnospiraceae_Tyzzerella*<br>*Firmicutes_Clostridia_Clostridiales_Lachnospiraceae_Lactonifactor*<br>*Bacteroidetes_Bacteroidia_Bacteroidales_Rikenellaceae_Alistipes*<br>*Bacteroidetes_Bacteroidia_Bacteroidales_Tannerellaceae_Parabacteroides*<br>*Firmicutes_Erysipelotrichia_Erysipelotrichales_Erysipelotrichaceae_Candidatus_Stoquefichus*<br>*Firmicutes_Clostridia_Clostridiales_Lachnospiraceae_XBB1006*<br>*Firmicutes_Clostridia_Clostridiales_Ruminococcaceae_Anaerotruncus*<br>*Actinobacteria_Actinobacteria_Bifidobacteriales_Bifidobacteriaceae_NA*<br>*Firmicutes_Clostridia_Clostridiales_Clostridiaceae_1_Candidatus_Arthromitus*

Figure S4. Venn diagram and the corresponding table depicting the number and classification of the genera that remained extinct at the time of and 6 months after the cessation of Abx treatment in young and/or older mice.
