# Supplementary material for: Dynamics of Gut Microbiota Recovery after Antibiotic Exposure in Young and Old Mice (A Pilot Study)
Source: Microorganisms. 2021 Mar 20;9(3):647. doi: 10.3390/microorganisms9030647 (PMC8003781; doi:10.3390/microorganisms9030647)
Supplement: Supplementary file 1 [file microorganisms-09-00647-s001.zip › Table S1.pdf]

Table S1. List of the most abundant genera in fecal samples of young and older mice.

Genera <u>decreasing</u> with age

Taxa	Average relative abundance in young mice	Average relative abundance in older mice	Fold change	statistical significance (DESeq2)
Candidatus_Stoquefichus	0.198%	0.000%	-1.00	
Candidatus_Arthromitus	0.147%	0.000%	-1.00	
Butyricicoccus	0.146%	0.000%	-1.00	
Harryflintia	0.092%	0.000%	-1.00	
Anaerotruncus	0.043%	0.000%	-1.00	
Ruminococcaceae_UCG-003	0.013%	0.000%	-1.00	
Lactonifactor	0.006%	0.000%	-1.00	
XBB1006	0.006%	0.000%	-1.00	
Ruminococcaceae_UCG-014	9.961%	0.302%	-0.97	
Lachnospiraceae_UCG-006	0.171%	0.007%	-0.96	
Ruminococcaceae_NK4A214_group	0.131%	0.011%	-0.91	
Lachnospiraceae_UCG-001	7.983%	0.940%	-0.88	
Anaerovorax	0.054%	0.012%	-0.78	
Ruminococcus_1	1.296%	0.300%	-0.77	
Ruminiclostridium_6	1.891%	0.478%	-0.75	
Ruminiclostridium	3.663%	1.094%	-0.70	
GCA-900066575	0.396%	0.142%	-0.64	
Lachnoclostridium	1.471%	0.561%	-0.62	
Roseburia	1.607%	0.638%	-0.60	*
Shuttleworthia	0.096%	0.039%	-0.59	
Parabacteroides	3.119%	1.279%	-0.59	*
Lachnospiraceae_FCS020_group	0.212%	0.088%	-0.59	*
Coriobacteriaceae_UCG-002	0.769%	0.323%	-0.58	
Lactobacillus	9.680%	4.150%	-0.57	*
Intestinimonas	0.913%	0.447%	-0.51	*
Family_XIII_UCG-001	0.090%	0.048%	-0.48	
Muribaculum	1.286%	0.745%	-0.42	
Oscillibacter	2.619%	1.529%	-0.42	*
Tyzzerella	0.143%	0.086%	-0.40	
Ruminiclostridium_9	1.592%	1.049%	-0.34	
Ruminococcaceae_UCG-005	0.335%	0.247%	-0.26	*
Bifidobacterium	8.621%	7.097%	-0.18	
Turicibacter	1.965%	1.715%	-0.13	
Ruminiclostridium_5	0.429%	0.388%	-0.10	
Alistipes	11.394%	10.648%	-0.07	*

Genera <u>increasing</u> with age

Taxa	Average relative abundance in young mice	Average relative abundance in older mice	Fold change	statistical significance (DeSeq2)
Desulfovibrio	1.106%	1.115%	0.01	
ASF356	0.703%	0.754%	0.07	
Acetatifactor	0.445%	0.494%	0.11	
Enterorhabdus	0.075%	0.084%	0.12	
Bilophila	0.154%	0.188%	0.22	
Ruminococcaceae_UCG-009	0.303%	0.381%	0.26	
Romboutsia	0.222%	0.446%	1.01	*
Lachnospiraceae_NK4A136_group	13.563%	27.392%	1.02	*
Ruminococcaceae_UCG-010	0.339%	0.794%	1.34	*
Clostridium_sensu_stricto_1	1.577%	3.716%	1.36	*
UBA1819	0.119%	0.312%	1.61	
A2	1.028%	2.695%	1.62	
Anaeroplasma	6.240%	16.851%	1.70	*
Lachnospiraceae_UCG-004	0.004%	0.018%	3.14	
Faecalibaculum	1.584%	10.256%	5.47	
