# Supplementary material for: Dynamics of Gut Microbiota Recovery after Antibiotic Exposure in Young and Old Mice (A Pilot Study)
Source: Microorganisms. 2021 Mar 20;9(3):647. doi: 10.3390/microorganisms9030647 (PMC8003781; doi:10.3390/microorganisms9030647)
Supplement: Supplementary file 1 [file microorganisms-09-00647-s001.zip › Table S2.pdf]

**Table S2.** List of the most abundant genera in fecal samples of young mice before and after 10-day broad-spectrum antibiotic treatment.

| Genus | Average relative abundance | |
|---|---|---|
| | Before Abx | After Abx |
| *A2* | 1.03% | 0.24% |
| *Acetatifactor* | 0.44% | 0.18% |
| *Alistipes* | 11.39% | |
| *Anaeroplasma* | 6.24% | |
| *Anaerovorax* | 0.05% | |
| *ASF356* | 0.70% | 0.66% |
| *Bifidobacterium* | 8.62% | 34.66% |
| *Bilophila* | 0.15% | 0.09% |
| *Butyricicoccus* | 0.15% | |
| *Candidatus_Arthromitus* | 0.15% | |
| *Candidatus_Stoquefichus* | 0.20% | |
| *Clostridium_sensu_stricto_1* | 1.58% | |
| *Coriobacteriaceae_UCG-002* | 0.77% | |
| *Desulfovibrio* | 1.11% | 0.34% |
| *Enterococcus* | 2.21% | |
| *Enterorhabdus* | 0.07% | 0.13% |
| *Erysipelatoclostridium* | 0.11% | |
| *Faecalibaculum* | 1.58% | |
| *Family_XIII_UCG-001* | 0.09% | |
| *GCA-900066575* | 0.40% | 0.12% |
| *Harryflintia* | 0.09% | |
| *Intestinimonas* | 0.91% | 0.58% |
| *Lachnoclostridium* | 1.47% | 0.48% |
| *Lachnospiraceae_FCS020_group* | 0.21% | 0.29% |
| *Lachnospiraceae_NK4A136_group* | 13.56% | 10.72% |
| *Lachnospiraceae_UCG-001* | 7.98% | 0.82% |
| *Lachnospiraceae_UCG-006* | 0.17% | |
| *Lactobacillus* | 9.68% | 35.86% |
| *Muribaculum* | 1.29% | 0.29% |
| *Oscillibacter* | 2.62% | 1.04% |
| *Parabacteroides* | 3.12% | 6.50% |
| *Romboutsia* | 0.22% | |
| *Roseburia* | 1.61% | 1.25% |
| *Ruminiclostridium* | 3.66% | 0.28% |
| *Ruminiclostridium_5* | 0.43% | 0.38% |
| *Ruminiclostridium_6* | 1.89% | 0.12% |
| *Ruminiclostridium_9* | 1.59% | 0.73% |
| *Ruminococcaceae_NK4A214_group* | 0.13% | |
| *Ruminococcaceae_UCG-005* | 0.34% | |
| *Ruminococcaceae_UCG-009* | 0.30% | 0.07% |
| *Ruminococcaceae_UCG-010* | 0.34% | |
| *Ruminococcaceae_UCG-014* | 9.96% | 1.60% |
| *Ruminococcus_1* | 1.30% | |
| *Shuttleworthia* | 0.10% | |
| *Turicibacter* | 1.97% | |
| *Tyzzerella* | 0.14% | |
| *UBA1819* | 0.12% | |
