# Supplementary material for: Dynamics of Gut Microbiota Recovery after Antibiotic Exposure in Young and Old Mice (A Pilot Study)
Source: Microorganisms. 2021 Mar 20;9(3):647. doi: 10.3390/microorganisms9030647 (PMC8003781; doi:10.3390/microorganisms9030647)
Supplement: Supplementary file 1 [file microorganisms-09-00647-s001.zip › Table S3.pdf]

Table S3. List of the most abundant genera in fecal samples of older mice before and after 10-day broad-spectrum antibiotic treatment

Genus	Average relative abundance	
	Before Abx	After Abx
A2	2.70%	0.40%
Acetatifactor	0.49%	0.16%
Alistipes	10.65%	
Anaeroplasma	16.85%	0.07%
ASF356	0.75%	0.56%
Bifidobacterium	7.10%	28.37%
Bilophila	0.19%	0.09%
Clostridium_sensu_stricto_1	3.72%	
Coriobacteriaceae_UCG-002	0.32%	
Desulfovibrio	1.12%	0.29%
Enterorhabdus	0.08%	0.06%
Erysipelatoclostridium	0.13%	0.72%
Faecalibaculum	10.26%	
GCA-900066575	0.14%	0.06%
Intestinimonas	0.45%	1.09%
Lachnoclostridium	0.56%	0.44%
Lachnospiraceae_FCS020_group	0.09%	0.55%
Lachnospiraceae_NK4A136_group	27.39%	9.43%
Lachnospiraceae_UCG-001	0.94%	4.14%
Lactobacillus	4.15%	39.71%
Muribaculum	0.74%	0.24%
Oscillibacter	1.53%	1.47%
Parabacteroides	1.28%	8.65%
Romboutsia	0.45%	
Roseburia	0.64%	0.72%
Ruminiclostridium	1.09%	0.24%
Ruminiclostridium_5	0.39%	0.39%
Ruminiclostridium_6	0.48%	0.23%
Ruminiclostridium_9	1.05%	0.74%
Ruminococcaceae_UCG-005	0.25%	
Ruminococcaceae_UCG-009	0.38%	
Ruminococcaceae_UCG-010	0.79%	
Ruminococcaceae_UCG-014	0.30%	0.85%
Ruminococcus_1	0.30%	
Turicibacter	1.72%	
Tyzzerella	0.09%	0.06%
